# Explaining the experiences of health care providers regarding organizational factors affecting health education: a qualitative study

**DOI:** 10.1186/s12909-022-03807-8

**Published:** 2022-10-27

**Authors:** Fatemeh Bastami, Fereshteh Zamani-Alavijeh, Iraj zareban, Marzieh Araban

**Affiliations:** 1 Social Determinants of Health Research Center, School of Health and Nutrition, Lorestan University of Medical Sciences, Khorramabad, Iran; 2 Department of Health Education and Health Promotion, School of Health, Isfahan University of Medical Sciences, Isfahan, Iran; 3 Health Promotion Research Center, Zahedan University of Medical Sciences, Zahedan, Iran; 4 Menopause & Andropause Research Center, Ahvaz Jundishapur University of Medical Sciences, Ahvaz, Iran; 5 Department of Health Education and Promotion, Public Health School, Ahvaz Jundishapur University of Medical Sciences, Ahvaz, Iran

**Keywords:** Health care providers, Health education, Qualitative study, Planning, Organizing, Monitoring, Evaluation

## Abstract

**Background:**

Health education is considered the most important component of primary health care. Paying attention to organizational factors can help to improve the quality of health education. Therefore, the present study was conducted to explain organizational factors affecting health education among health care providers.

**Method:**

This is a qualitative, descriptive, and phenomenological study that was conducted between 2020 -2022 on 50 health care workers who had been selected by purposeful sampling method in different settings including hospitals, GP office, behavioral disease counseling center, universities, and comprehensive health centers in the south and the center region of Iran. Data were collected by in-depth, semi-structured, and individual interviews, as well as focus group discussion, and continued until data saturation. Data were analyzed by MAXQDA software using qualitative content analysis in three stages: preparation, organization, and reporting. To evaluate the scientific accuracy of the findings in this study, 4 criteria of Lincoln & Guba were used.

**Results:**

The results revealed that every practice and policy in a health care organization from assessing needs, setting goals, planning activities, implementations and measurement outcome could affect health education practice and subsequently the health of population; nevertheless, the crucial role of health education practice is being neglected in health organizations. Organizational factors affecting health education were classified into three categories of planning, organizing, and also monitoring and evaluating. The category of planning had three subcategories of infrastructure planning, manpower planning, and design and planning for implementation of health education programs. The categories of organizing had two subcategories of coordination between different units of the Minister of Health and coordination between the health sector and other sectors of society. The categories of monitoring and evaluation had three subcategories of proper feedback, bureaucracy system, reward or reinforcement, failure to define health education as part of the job description, and the impact of electronic health records on the quality of evaluation.

**Conclusion:**

The results offer expertise and preliminary tools to help with evidence-based health education program planning and evaluation. The Support of managers, like providing manpower in accordance with the target population and infrastructure, can improve health education in the health system. In addition, intra- and inter-sectoral coordination at different levels of the health system to implement tailored programs according to the needs of clients by health education professionals, and the use of health education theories seem necessary. It is also recommended to review the monitoring system with appropriate feedback, define health education as a healthy activity, and develop appropriate criteria for better implementation of health education.

## Introduction

Health education is considered the first and most important component of primary health care. Perhaps there has never been a time to facilitate behavioral changes to such an extent as there is now, because of the great demand for health education. Health education, as the most pivotal step to achieving positive health and preventing various diseases, helps people to deal with health problems, make appropriate decisions and change behavior in the face of problems [1, 2]. Health education is one of the most important parts of health promotion provided by health care providers, which aims to influence the behavior of people attending health centers, through formal and informal activities. Clients always want health care worker to answer their questions with full knowledge [3, 4].

Phenomena such as bacterial resistance to antibiotics, genetic alterations of pathogens, and the emergence of new diseases such as covid infection have questioned the idea of a future without communicable diseases [5]. On the one hand, due to changes in living conditions, we are witnessing the emergence of dangerous pandemics, which if not controlled, can reemerge with greater intensity in the future. On the other hand, despite advances in biomedicine and its exorbitant costs, the mortality rate and life expectancy have not changed a great deal in developed countries [6]. Also, today, pessimism about medicine has grown and medical science has been gradually questioned and criticized due to its inability to treat some diseases such as cancer and AIDS. These bitter facts have placed a heavy burden on the health system, and the importance of appropriate structures to prevent health crises, deadly pandemics and, if occurred, rapid response to them have become more prominent than ever [7, 8]. Health education and health promotion activities have the greatest potential to reduce disease, mortality, and staggering medical costs. They also are the best hope for long-term improvement in the quality of life of the population [9].

Implementation of patient education is always accompanied by obstacles and facilitators. Studies show that the existence of numerous barriers alters the effectiveness of health education. Barriers to health education refer to deterrents that limit the ability of health care professionals. Previous studies have been examined the factors related to health educators, such as inadequate communication skills, lack of knowledge about clients' right to education, insufficient caregiver’s knowledge and skills, caregivers' disbelief about health education as one of their professional duties, not valuing health and professional education, perceived self-efficacy of health educator, etc. [10–14].

In addition to identifying the factors related to a health educator, paying attention to the organizational factors that facilitate education can help to improve the quality and quantity of health education. A previous systematic review of studies in different countries of the world showed that organizational factors including high workload and lack of time and resources are among the factors that affect preventive health promotion activities [15]. Previous studies in Iran have referred to factors such as organizational culture, health care system management, the physical environment, and working conditions, an insufficient number of employees, and lack of time as barriers to patient education [16, 17]. However, so far no study in Iran has examined all organizational factors despite their interactions with health education. Due to cultural differences and differences in health care delivery systems in different countries, it is necessary to study this issue in each country. Despite extensive study of previous texts and studies, at least in Iran, no study has deeply explored the nature of organizational factors affecting health education, and this issue has been less studied in its true context, through the real experiences of health care providers. For this reason, sufficient knowledge about the organizational factors affecting health education in comprehensive health centers and hospitals has not yet been obtained. Therefore, the main question is which organizational factors affect health education and how effective are they? Since health education is a complex subject and is related to social, environmental, and psychological issues, the most appropriate approach to better understand its dimensions is the qualitative method with an inductive approach. Therefore, the present study was conducted to explain the organizational factors affecting health education among health care providers.

## Materials and methods

### Design

This is a qualitative, descriptive, and phenomenological study that was conducted to explain the experiences of health care providers regarding organizational factors affecting health education in Iran. This study was a continuation of a previous study [3].

### Participants and settings

The whole sampling of this project has been done from 2020 to 2022. This work applied inductive approach to understand organizational factors affecting health education practice. This study was conducted on 50 health care workers who had been selected by purposeful sampling method in different settings including hospitals, GP office, behavioral disease counseling center, universities, and comprehensive health centers in the south and the center region of Iran. The participants consisted of nurses (*n* = 11), midwives (*n *= 5), social pathologists (*n* = 3), people with master's degrees in health education (*n* = 6), public health providers (*n* = 15), general psychologists (*n* = 5) and people with a Ph.D. degree in health education and health promotion (*n* = 5) who had an average of 6 years of experience in the health centers. Inclusion criteria were; having at least one year of work experience in comprehensive health care centers and hospitals, current or previous experience of health education in health care system, and being interested in expressing personal experiences. Exclusion criteria included; unwillingness to continue participating in the study and inability to express personal experiences clearly (Table 1).

**Table 1:** Demographic characteristics of the participants

Variable		Quantity	Percentage
**Gender**	Woman	29	58
	Man	21	42
**Professional filed**	Public health	15	30
	Midwifery	5	10
	Nursing	11	22
	Health education	11	22
	Social pathology	3	6
	psychology	5	10
**Education**	Bachelor’s degree	36	72
	Master’s degree	6	12
	Doctoral	8	16
**Service delivery center**	Hospital	10	20
	GP offices	5	10
	Comprehensive health centers	15	30
	Behavioral disease counseling centers	9	18
	Universities	11	22

### Data collection

In qualitative studies, although the researcher designs the study and analyzes the data, he or she is also a data collector. In this study, the researcher established close and direct communication with the participants to obtain valid and real information. Data collection was performed using semi-structured, in-depth, and individual interviews as well as 4 focus group sessions. Data were collected only through audio recording. In order to participate in the interviews and focus groups, people were free to choose to participate in individual interviews or focus groups, so they participated in one of them based on their convenience.

### Interviews

Twenty nine individual interviews were conducted. Individuals were interviewed only once. Their participation was not repetitive. The interviewees did not participate in focus groups. First, for icebreaking, the importance of the role of health education in providing health services was discussed, and the objectives and method of the study were explained, followed by the main interview questions, and all participants were asked to express their experiences, perceptions, and feelings. Finally, they were asked to make suggestions for improving the health education’s role in providing health services.

The interviews began by asking the participants to introduce themselves and give a history of their work experience. Initially, they were asked what organizational factors they think are the obstacles and facilitators of health education. They were then asked to talk freely about their experience in providing health education. According to the participants, the interviews continued with phrases such as; "can you explain more?" or "how did you conduct that training session"? The duration of each interview lasted between 20 and 60 min, depending on the participant, the situation, and the discussion process. Interviews continued until no new data was obtained.

### Focus groups

Besides individual interviews, the focus group was also used to enrich the data. Some people are probably more comfortable talking in the focus group than when they are interviewed in person and tend to share their experiences with their peers [18].

Twenty-one people participated in focus groups. Individuals participated in the focus group only once. First, for icebreaking in three focus groups with 6–8 people, the role of health education in providing health services was discussed and the objectives and method of the study were explained; then, the main interview questions were asked and all participants were asked to express their experiences, perceptions, and feelings. Finally, they were asked to make suggestions for improving health education’s role in providing health services.

Two focus groups were held in the counseling room of the comprehensive health centers and 1 in the nurses' restroom in the hospital. Each focus group session lasted approximately 45–60 min. In all focus groups, the same general questions about experiences in providing health education (ie; talking about successful and unsuccessful experiences in providing health education to clients, and what solutions did they use to solve the problems and remove the obstacles?) were asked to the participants. Although some of the questions were similar, people cited different experiences because participants included different occupations, including a nurse, midwife, social pathologist, general psychologist, public health expert, master’s, and Ph.D. in health education and promotion. So people came up with different experiences, and these different experiences led us to try different probes to get richer data.

Subsequently, the probing questions were used to further discuss the topic. Different probing questions were used in different groups, depending on the responses given by the participants.

### Data analysis

The data analysis was conducted simultaneously with data collection, using the qualitative content analysis method, which was performed in three stages: preparation, organization, and reporting [19]. In the preparation stage, each audio file was considered as a unit of analysis. First, the audio files of each interview were immediately typed verbatim. Also, the audio files were listened to several times and the texts were read repeatedly to give the researcher a general understanding of the data. Initial coding was used to organize the data. Also, using the inductive approach, the analysis of data was continued by extracting semantic units from each text line and conducting initial coding. The coded data were then recorded in the code sheet for later reference. Classification of the codes began after the first few interviews. The codes were classified according to their similarities and differences. By repeating the mentioned process for each interview, some new topics were added until the final pattern emerged. Merging and comparing groups reduced the number of categories. Subcategories were also formed based on similar features and showed the names of categories [20, 21]. Data were analyzed by Maxqda software.

### Scientific accuracy of the findings

To evaluate and ensure the scientific accuracy and validity of the findings, the criteria proposed by Lincoln & Guba quoted by Polite were used [22–24]. To ensure the validity of the findings, the researcher took sufficient time to collect the data and maintained a long-term engagement by reviewing the data and continuously communicating with the target group to gain a better understanding of them. For further validation of the extracted content, several coded interviews were returned to the participants for review to confirm the correctness of the researcher's interpretations of their statements. The reliability of the findings was assessed through peer review and member check methods. To ensure confirmability, the researcher tried to avoid any presumptions in the process of data collection and analysis.

## Results

The experiences of the health care worker about the organizational factors affecting health education were categorized into 3 main categories, including planning, organizing, and monitoring and evaluation (Table 2).

**Table 2:** Organizational factors affecting health education among health care personnel

N	**Categories**	**Sub-Categories**				
1	Planning	Planning related to the provision of appropriate infrastructure	Insufficient tools and equipment Lack of suitable space The effect of electronic records on the quality of health education			
		Planning related to the hiring of adequate and appropriate human resources	The disproportion between the number of staff and workload The hiring of educated personnel to take on multi-professional roles Lack of job security			
		Planning related to the design of appropriate health education programs	Design of programs that lead to the failure of health education	The disproportion between the subject and content of the educational program, and the age and needs of clients Design of personal educational programs without considering the needs of family and society Design of the program of using health ambassadors to educate the community without considering the infrastructure		
			Design of programs that lead to the success of health education	Design of creative educational materials		
				Designing a national self-care program	Designing individual training program	Issuing patient education card
						Training in follow up program
					Designing group education programs	Holding group education program
						Providing education and attracting the participation of peers
						Using health volunteers program in community education
2	Organizing	Coordination between different units of the Ministry of Health Coordination between health sectors and other sectors of society				
3	Monitoring and evaluation	Bureaucracy system Rewards and reinforcements Failure to define health education as part of the job description The impact of electronic health records on the quality of evaluation				

### Planning

This category refers to the managers’ planning for infrastructure, manpower, and the design of health education programs.

### Planning related to the provision of appropriate infrastructure

This category points to the inadequacy of hardware and software environments for the implementation of health education. This category has 3 sub-categories of insufficient tools and equipment, lack of suitable space, and the impact of electronic health records on the quality of health education implementation.

### Insufficient tools and equipment

This concept shows that it does not make sense for people to be given health education but to be abandoned. The health system is responsible for the training it provides and must make arrangements for it in the system. The lack of necessary equipment in the environment causes the client not to follow the training provided. One of the midwives working in the comprehensive health centers stated: "*When I teach, some facilities should be provided…For example, I teach women to have a Pap smear, so there must be a place to provide the smears at a low price for them*."

### Lack of suitable space

According to the results of the present study, health care providers are multidisciplinary and therefore provide health services to different groups in the community. Due to the lack of workspace, several health care providers provide health services in a common room, so there is no privacy for clients to consult and provide health education services. Clients do not even have the comfort of asking some questions of health care providers.

Limitation in the availability of suitable space leads to a lack of privacy to provide health education and counseling to clients, so essential training is ignored due to the lack of a safe physical environment. A public health provider in this regard stated: "*A pregnant woman comes for health care and at the same time another woman comes with her husband to take care of her child. So this pregnant woman does not feel safe when we teach her. Even if we asked her when her last LMP was, she will be so embarrassed to answer us. She can't even roll up her sleeves so we can take her blood pleasure*."

### The impact of electronic health records on the quality of health education

Registration and provision of primary health care to clients in Iran are done through the **Sib** system. This system is part of the electronic system of health records, and despite its advantages, it still has drawbacks. For example, some care options are not properly defined for some population groups and require revision. In addition, the slow speed of the Internet when working with this system has disrupted the provision of care. This slowness and disconnection of the Internet have a devastating effect on the continuity of communication between the client and the health care provider.

According to the experience of the health workers, completing a health record electronically will cut off the face-to-face communication between the health care provider and the client, and reduce the quality of counseling and training. Health care workers attributed this to a lack of infrastructure. A public health provider stated: "*The internet is constantly cutting off and its speed is very low. You are completing the file and giving some training at the same time, and all of a sudden the internet is cut off and nothing can be done about it*."

### Planning related to the hiring of adequate and appropriate human resources

According to the findings, there are four challenges regarding the supply of manpower, including disproportion between the number of staff and workload, hiring of educated personnel to take on multi-professional roles, and inadequate job security for health care workers.

### The disproportion between the number of staff and workload

According to the present study, when the number of clients or patients to receive services is high, the health care provider or nurse can not devote time to training all of them and can only provide health services without sufficient training. Lack of manpower and high workload affect the quality and quantity of health education. A nurse in this regard said: "*The number of patients does not always match the number of staff. The staffs in the hospital have to educate up to ten patients at the same time, and patients who are in pain cannot wait at all*." A public health provider stated: "*First they said a health worker for every 2000 people, but now there are 3,000 to 3,500 people for every health worker, and they are constantly reducing the manpower*."

### The hiring of educated personnel to take on multi-professional roles

This is a challenge for staff who have studied in a particular field, and are only interested in providing services in that field. According to the participants' experience, this affects both the quality and quantity of training provided. A public health providerin this regard stated:" T*he workforce like midwives easily leave the system because they do not want to teach health to all groups. Their interest in their profession prevents them from being active in other areas*."

The second challenge related to manpower is the multidisciplinary functionality of healthcare workers in comprehensive healthcare centers. This issue causes a lack of concentration in the trainer and a lack of time to provide health education. A *midwife* stated: "*I am a midwife and I have to work as a midwife, but I am told to vaccinate people and see elderly people, and if I want to teach about Pap smear or breast cancer, I have no time because someone comes to the table and ask me to give him/her a vaccine or an elderly person sits on a chair and asks me to check his/her blood pressure. We can only give general education and just an overview of the topics*. "

### Inadequate job security

Another challenge related to the supply of manpower is inadequate job security for health care workers, which affects the quality of health education.The present study showed that the employment status of some health personnel was still unclear despite at least three years of experience, and it was possible that the health system would ask them to leave the system and be fired.

"We are constantly told about redundancy. I am a mental health expert, but I am constantly stressed. How should I pay attention to my work or the way I train? Every morning I say to myself that today, they are going to tell me that I have been redundant."(general psychologists).

### Planning related to the design of appropriate health education programs

Participants listed the programs they have been responsible for designing or implementing and said some of them have been successful and some unsuccessful. These programs can be divided into two categories.

### Design of programs that lead to the failure of health education

#### The disproportion between the subject and content of the educational program, and the age and needs of clients

According to the findings of the present study, health education programs are successful when they are tailored to the needs of population groups. One of the principles of health education is to create motivation and interest in the target group, and this happens when health education meets their needs.

The disproportion between educational programs and clients is one of the obstacles to effective health education, which indicates the inefficiency of health systems’ planning. An official of county health education stated: "*Sometimes we are forced to teach a subject that is not appropriate to the age and understanding of clients, such as cancer education for someone with elementary school education*."(Master's degree in health education).

#### Design of individual educational programs without considering the needs of family and society

Although individual training may increase people’s awareness and understanding, and even change their attitude, if not supported by community members, especially the families, it cannot change behavior and improve health, and may even cause unresolved conflicts between a person and others. This is because people are influenced by abstract norms such as family and close people. One participant in this regard stated: "*We usually see clients in isolation, we say something to them and their families say something else to them and this creates a contradiction and challenge from them … It means that, when we train someone and give him/her more responsibility, he/she later tells us; I wanted to follow your instruction, but I got into an argument with my partner because he/she does not agree with that*."(Ph.D. in health education).

#### Design of the program of using health ambassadors to educate the community without considering the infrastructure

A family health ambassador is a family member who voluntarily takes responsibility for his or her health and that of his or her family members. After completing training courses (in person or online) in the field of self-care, the health ambassador transfers his or her knowledge to his or her family members. The health ambassador can control minor and chronic illnesses of himself and his family members. This reduces the family's need for multiple visits to GP or emergency room.

This program has had some challenges in providing health education, and the participants shared their experiences in this field. "*Every family that wants to select a health ambassador must pay attention to special conditions that the health ambassador should have. For instance, he or she must have at least secondary education, and must pass the educational materials to the family. …. We have many problems in this field, for example in the village, most people have primary education and do not have internet access and even if some places do have internet access, they do not know how to use it. In addition, an application should be uploaded to the ambassadors' smartphone, so they must be always online to receive the educational contents and pass them on to family members … pieces of training are mostly virtual.*"(Master's degree in health education).

#### Design of programs that lead to the success of health education

The findings of the present study showed that taking measures in health education leads to the success of health education programs. These arrangements emerged as concepts including the creative design of educational materials and the design of a national health self-care program.

#### Design of creative educational materials

Providing educational materials such as pamphlets and booklets to clients increases the effectiveness of face-to-face or group training. But what is important is to use creativity in the preparation of educational materials that will attract the attention of clients and make them more willing to learn the educational content. A nurse in this regard stated: "*We have few specialist physicians in our city that people trust, and are considered as references …. We prepare the educational contents with the photos of them and distribute them among people*."

#### Design of a national self-care program

Self-care is the practice of using knowledge, skills, and abilities as a resource to take care of own health independently. This category emerged with two subcategories, including the design of individual training programs and the design of group training programs.

#### Design of individual training programs

In the present study, two concepts emerged regarding the design of individual training programs, including the issuance of patient education cards and training in the follow-up program.

#### Issuing patient education card

This category was one of the aspects of the national self-care program. This concept refers to the use of educational aids in providing health education, which leads to the success of health education programs. In this regard, the participants ingeniously prepared patient education cards so that, when patients get discharged from the hospital could receive the necessary training to take care of themselves at home. The content of these cards was brief and they had been prepared with very simple sentences. This created an opportunity to provide health education, especially for patients with chronic diseases. A nurse in this regard stated: “*We have created a patient education card for people with chronic diseases such as high blood pressure or diabetes who have been discharged from the hospital. This card is the size of an A5 paper, in which patient details, file number, recommendations, and time of next appointment are written on one side, with some brief instructions on the other side. For example, we write; in order not to infect the wound, you should do this. We also write the phone number of our ward, just in case they have any questions. The sentences are very simple so that patients with an eighth-grade education could understand them*."

#### Training in the follow-up program

The patient follow-up program is one of the programs that have been successful in implementing the self-care program, especially for people who have been discharged from the hospital. This program is implemented in hospitals that have allocated funds for health education in their accreditation.This concept shows that the allocation of funds for health education in health settings causes health personnel to implement it.

The participants shared their successful experiences in this field. A nurse who was in charge of health education in the hospital said: "*Nurses read the discharge lists and find patients’ phone numbers to call them and ask questions about complications such as nosocomial infections, urinary incontinence due to catheterization, and their next appointment time. Sometimes patients have a new question, so they call us from home and ask us their questions. The staff in each shift spend 30 to 60 min on this. With this program, people's satisfaction has increased significantly and they feel that they are receiving the services at their house*."

#### Design of group training programs

The participants believed that group training is more effective and useful in some subjects than individual training. These pieces of training include holding group training sessions, providing education and attracting the participation of peers, and using the health volunteers program in community education.

#### Holding group training sessions

According to the principles of health education, holding group sessions for individuals is one of the facilitators of health education, because it allows group members to interact with each other and share their experiences. As a result, the impact of training will be more stable. A participant in this regard stated: "*Group training is much more effective because, in a group, people ask questions, so the learning becomes participatory and the pieces of training stay in learners’ minds for longer. For example, I teach the danger signs of pregnancy to pregnant women individually, and the next month when one of them comes back and I ask her about the danger signs of pregnancy, she answers as if she knows nothing but the danger signs of pregnancy*."(Master's degree in health education).

#### Providing education and attracting the participation of peers

This study showed that when patients receive health education from people who are in similar health conditions, they follow their recommendations more**.** In peer education, a health care worker or nurse trains several people and motivates them to pass on organized educational activities to their peers, who are similar in age, background, and interests. A nurse in this regard stated: “*We write down the names and phone numbers of diabetic patients. We also invite them to the hospital once every three months to receive training from two or three of them who have been trained by us… For example, someone says I have had diabetes for two or three years and when I take this medication, my blood sugar stays in the normal range, or when I follow my diet and take my insulin, I no longer have leg ulcers. Hearing such information from peers is more effective. This way, we only work with those few people and they pass on our training to others*."

#### Using health volunteers program in community education

According to the present study, when health education is transmitted by one of the people in the region with whom they share a common language and culture, it is more welcomed. By educating health volunteers, we can educate all people in one area, said the participants. A participant referred to the benefits of this program and stated: "*The health volunteers are outside the health system, and are often local people who have cultural similarities with the people of the region. They have a high school diploma. We teach them the educational contents in simple language and they pass them on to their families and friends*."( Ph.D. in health education).

## Organizing

Organizing is the process by which, work is divided between individuals and workgroups, and is also coordinated between them to achieve the set goals. This category had two subcategories of coordination between different units of the Ministry of Health and coordination between the health sector and other sectors of society regarding health education.

### Coordination between different units of the ministry of health

This concept refers to the usefulness of a committee consisting of specialists in health system units, including infectious diseases, non-communicable diseases, elderly health, child health, adolescent health, reproductive health, middle-aged health, and so on. Before producing and publishing health education content, it is first reviewed by this committee. This makes health education integrated and coordinated with all health units.

This concept refers to the role of health education in creating intra-sectoral coordination among health technical units such as family health, non-communicable diseases, etc. By creating this coordination, health education services will be provided in a structured way for each component of primary health care. "*Every medium, like pamphlets, posters, and teasers, has its standard that must be considered in the production process. Before, each technical unit was responsible for determining what media to produce independently. This is while the responsibility of the health education unit is to supervise the production and distribution of all educational media. We have established a deeply structured committee that the financial unit cannot afford to pay for the media without the approval of the health education unit*."(Master's degree in health education).

### Coordination between the health sector and other sectors of society

The participants believed that cooperation between organizations and institutions outside the university regarding health education is necessary and if they cooperate, the implementation of health education will be facilitated and will cause people outside the health organization to play a role in the implementation of health education. This makes educational products to be available to a larger number of people. One participant said: "*A good thing is that there is a link between the health system and other organizations, such as the governor's Basij. They gather their employees to train them. For example, the municipality has a cooking program that invites our nutritionist to talk about healthy eating*."(Ph.D. in health education).

### Monitoring and evaluation

From the participants' point of view, problems related to monitoring and evaluation methods were obstacles to improving the quality of training. This category had 5 sub-categories how to provide proper feedback to staff, bureaucracy system, reward or reinforcement, failure to define health education as part of the job description, and the impact of electronic health records on evaluation.

### How to provide proper feedback to staff

Participants referred to the monitoring and evaluation that have been limited to completing forms, and the lack of appropriate feedback, which demotivate the staff. A participant about not getting proper feedback stated that: "*Clients should evaluate the training. For instance, the mothers who come to receive service should be asked whether they have received training on a particular topic from the staff or not. Then, the fee must be given to the health educator*”(A public health expert). Another participant regarding improper feedback from managers stated that: “*They should obtain real feedback and propose solutions, not just blame us*”(A public health expert).

### Bureaucracy system

A bureaucratic system refers to limiting the provision of appropriate feedback to assessment staff to document recording or quantitative assessment. This concept shows that monitoring should be done in various ways, such as checking the satisfaction of clients receiving health education services, and not just reviewing documents. Most health workers believed that educational documentation alone could not reflect the entire educational activity. In this regard, a public health provider said: "*Our system is bureaucratic, and no one comes to check the quality of training, whether group or face-to-face. They do not tell us to give high-quality training*."

The health care workers said that not all pieces of training could be recorded. Despite doing the training, it is not possible to properly record and document it due to a lack of time to register for the training. From the participants’ viewpoint, the long bureaucracy in monitoring and evaluation has reduced the quality of evaluation in the system. A public health provider stated: "*We do a lot of paperwork; I mean it is not like we inject vaccines and train patients and that’s it. After all that, we still have to spend lots of time documenting them all in the file. Therefore, the quality of our work decreases*."

### Reinforcements

Managers' emphasis on implementing health education and material or spiritual appreciation of active personnel increases their motivation. A health education official said: "*I wrote an incentive for all health education officials of environmental center and gave them a certificate of appreciation that had spiritual value. I give them a certificate every year because it has an administrative impact on their promotion. Then, the heads of one of the centers said, the management told me to introduce 10 people from each center who have been active in this regard every month, so that I can also give them overtime pay. I saw it worked*."(Master's degree in health education).

### Failure to define health education as part of the job description

Health education is not defined as part of the duties of health care personnel, and no budget has been designated for it in the accreditation of health organizations. However, if it would be defined as part of personnel’s duties, its role in the effectiveness of primary health care will be further emphasized. A nurse in charge of a hospital's health education said: "*Even in the accreditation guide of health-promoting hospitals has not been mentioned that patient education is one of the duties of nurses and they are obligated to do that. The health-promoting hospitals should also have an operational plan for it, like exactly what is done in ordinary hospitals*."

### The impact of electronic health records on the quality of evaluation

Electronic health records, despite their many benefits, have had detrimental effects on monitoring and evaluation. Evaluation is based on the level of service delivered. This has led to a reduction in service delivery instead of control over the quality of services. One participant in this regard stated: "*With the electronic health records, evaluation has become quantitative and not qualitative. This has caused health care providers to document false records. When they tell us why the level of care is poor for the elderly and mothers, whether we like it or not, we are also moving towards quantity. This way, anyone who has the national number of a family member can document anything by it, so no proper training will be provided*."(Ph.D. in health education).

## Discussion

Three main categories including planning, organizing, and control emerged in this study that aimed to explain the experiences of health care workers about organizational factors affecting health education. According to previous texts, these three concepts are important and key duties of management in any organization. Management is the coordination of human and material resources to achieve organizational goals.

Planning is the first and most important activity necessary to achieve the desired results. Creating the desired situation in the future and finding ways and means to achieve it is planning [25]. This category had three subcategories planning for infrastructure, manpower, and design and implementation of health education programs.

The infrastructure consists of both hardware and software environments, including the sufficiency of equipment, suitable space, and the impact of electronic health records on health education promotion. Participants stated that the reason for not providing health education or the lack of individuals’ adherence to training provided is the lack of facilities and equipment such as kits and space for both individual counseling and group training. In the previous study, the physical environment, including space and appropriate equipment for health education, also had priority [26]. Even when training is vital, it is not provided due to a lack of adequate space. The social stigma of diseases in Iran, such as depression, is one of the most important reasons for privacy that should be considered in providing education and counseling [27].

One of the most important challenges regarding human resource is the high workload and miss match between the number of staff and the number of the target population. In a previous study, heavy workloads and lack of sufficient time to implement health education were mentioned as the most important barriers to patient education [28]. In the present study, multi-occupational health workers believed that a lot of time is spent on various primary health care processes such as caring for mothers, the elderly, etc., and this leaves the staff with not enough time to hold individual and group training. Providing sufficient manpower in accordance with the population covered by comprehensive health centers ensures that, staffs have sufficient time to provide health education services.

Health education programs refer to the planned design and implementation of group and individual education. Adherence to the training provided may be challenging if it conflicts with the norms of clients. Therefore, the best strategy is to hold joint classes for the person and his significant others. In previous studies, educating people who influence one's beliefs has been highlighted, which is at the heart of the theory of planned behavior and social cognition theory [29, 30]. In addition, this measure may be able to cover the shortage of manpower in the field of health education to some extent. This way, health education professionals select people from the target community and train them so that they can pass on the training to others. The participants also referred to the mismatch of compulsory education with the level of understanding of trainees. In this regard, Bloom’s taxonomy talks about the need to provide education at the level of trainees’ understanding [31]. This issue should be carefully considered by the officials.

The national self-care program focuses on health education programs that require proper planning. This program has created challenges and opportunities for the health system. Although self-care is an activity that people take to maintain and improve their health, sometimes this care is extended to their children, friends, and neighbors [32]. Realization of the slogan: "Health for the people and by the people" requires people’s participation as one of the principles of health education, which enables communities to learn healthy practices [33]. In this regard, various programs have been developed around the world to promote health, including the health volunteers program [34], and health ambassador program in recent years [35], which is one of the self-care programs in macro-health policies. The findings of Zareipour et al., show that a self-care program with a focus on self-care during COVID-19 raises the awareness of the health ambassadors and these ambassadors pass on the training to their family members and friends [35].

One of the challenges in this area is the type of people's participation in sessions held by health ambassadors and vice versa. Organized participation in the field of health care requires special infrastructure, attitudes, and skills that will increase the capabilities of health organizations. Based on the results of the present study and in accordance with the preceding proceed model, to implement the health ambassador program successfully, we need predisposing factors such as literacy, knowledge, and attitude; enabling factors such as internet access and smartphone; and reinforcing factors such as support system [36]. A study showed that empowering members of the village Islamic council as health ambassadors who have been elected by the villages will increase people's participation in health systems and especially, in self-care programs dynamically and effectively [37].

One of the organizational facilitators of health education in Iran’s national self-care program is the health volunteers program. In the Nepalese, a voluntary community scheme was launched. After a few years of volunteering, despite poor support and management of the project, there was a significant improvement in prenatal care, vaccination coverage, ORS use, and nutritional status of children under 5 years of age [38]. A study was conducted in Iran to investigate the relationship between the existence of the health volunteers and the use of primary health care, and the results showed that children and women who were working with health volunteers used primary health care significantly more than those who did not [39]. According to previous studies conducted in Iran, despite the effectiveness of health volunteers in the promotion of health indicators, numerous health volunteers in recent years have stopped working with comprehensive health centers. Factors such as expectations from the health volunteers when selecting them, the need to use experienced trainers, holding meetings between volunteers representatives and officials to express problems and provide solutions, holding appreciation ceremonies, and providing a suitable ground by attracting financial resources to support the health volunteers program can increase the participation of the health volunteers in the program and effectiveness of health volunteers program [40].

Other personal self-care programs such as patient education cards and training in follow-up programs that emerged in the present study have been the initiatives of health education managers in hospitals, which proved to be successful measures in providing health education. Previous studies show that organizational planning for the material and spiritual support of these programs makes them sustainable.

Health and education are complex issues and it does not make sense that only one of them can cause cross-sectoral coordination and improve service delivery [41]. This was evident in the participants’ statements. Involving non-health sectors in the health impact assessments (HIAs) can help to improve outcomes. In this regard, strategies should be designed that enhance the effects of both education and health [42]. The involvement of other ministries, such as the Ministry of Education, has also been mentioned to improve basic education in other countries [43, 44]. Emerging diseases such as Covid-19 have highlighted the importance of cross-sectoral coordination [45].

One of the most important organizational factors highlighted by the participants was the inadequacy of monitoring and evaluation. Unfavorable feedback on the lack of qualitative evaluation has been mentioned by the authorities. Health officials call for staff training, but there is no quality control over how training is conducted. Proper feedback is not done. The function of managerial evaluation is to facilitate professional growth and improve the quality of education. A previous study has also referred to lack of control and supervision by managers as the most important obstacle to education [46].

The presence or absence of adequate rewards is another challenge of monitoring and evaluation in the health system. Employees, who do not have the support of authorities, engage in training activities with less motivation and derive. Lack of emotional support hurts employees' morale and performance and causes frustration, loss of self-esteem, and avoidance of educational interventions [47]. Previous studies have also addressed issues such as wage disparities, lack of organizational support, devaluing of health education, and emphasis on prevention in the health system [48, 49].

Bureaucracy refers to the documentation registration system, which is presented as a challenge for the health system in this study. Managers' evaluation of health workers' training activities is limited to training documents prepared by the staff themselves. This way of evaluation causes managers to pay too much attention to the quantity of training and no attention to the quality of training provided [50]. Most of the participants believed that training documentation alone could not reflect the actual training activity. In addition, due to a lack of time and skills, some training activities are not recorded. Previous studies have also referred to the lack of accurate and correct criteria in the evaluation as one of the reasons for the ineffectiveness of education, which is consistent with the results of the present study. Quantitative and inadequate monitoring and evaluation have been considered in other studies as effective organizational factors in health education [51].

### Strength and limitation

The most important of the present study is its qualitative nature because the data is explained by the participants without any presuppositions. The validity of the data was confirmed by the researcher's long-term involvement with the data (from 2020 to 2022), and review of the data by participants, colleagues, and research team members. Also, the principle of maximum variation during sampling, such as conducting research in the three major provinces of Iran, and on two sexes with different work experience, education, and fields of medical sciences in health centers, was observed in this study. Failure to interview high-level managers of the health system is a limitation of this study that should be considered in future studies.

All in all, the results revealed that everything in a health care organization from assessing needs, setting goals, planning activities, implementations and measurement outcome could affect health education practice and subsequently the health of population. Design, implementation and monitoring and evaluation of proper health education programs require organizing the components involved in health education and coordination of both internal and external sectors to ensure that, health education is implemented on a larger scale and more people have access to the education. Also, the result of the evaluation should be given to the health personnel and if needed, necessary educational workshops with models and theories of health education should be designed and implemented for them to increase their knowledge and abilities.

## Conclusion

Organizational factors affecting health education are in fact, the support of organizational management, which includes three important aspects of management, such as planning, organizing and monitoring, and evaluating health education. Managers' attention to proper planning in the provision of infrastructure and adequate manpower is important. However, the proper design of health education programs by health education professionals may be able to cover some of the shortcomings in this area. the results offer expertise and preliminary tools to help with evidence-based health education program planning and evaluation.

## Data Availability

The datasets used and/or analyzed during the current study are available from the corresponding author on reasonable request.

## Abbreviation

GP: General Practitioner
